# Is the Assessment of the Non-Paretic Lower Limb in Patients After Stroke Important When Planning Rehabilitation?

**DOI:** 10.3390/s25041082

**Published:** 2025-02-11

**Authors:** Agnieszka Wareńczak-Pawlicka, Przemysław Lisiński

**Affiliations:** Department of Rehabilitation and Physiotherapy, Poznan University of Medical Sciences, 60-545 Poznań, Poland

**Keywords:** stroke, non-paretic lower limb, ipsilateral side, ipsilesional side, wireless sensors, muscle strength, proprioception, range of motion, balance

## Abstract

(1) Background: Hemiparetic patients after stroke have deficits on the side of the body opposite to the brain lesion. The aim of this study is to assess the occurrence of deficits in the ipsilesional lower limb. (2) Methods: Twenty-nine stroke patients (SG) and 29 healthy volunteers (CG) were recruited for this study. Passive (PROM), active (AROM), fast range of motion (FROM), and joint position sense (JPS) in the knee joint were measured using a wireless motion system. Participants were also assessed using the step test, balance platform, and the isometric protocol of measuring the strength of the extensor and flexor muscles of the knee. We compared non-paretic lower limb outcomes to the paretic side and a control group. (3) Results: The results showed a difference between the results of the ipsilesional side of the body of stroke patients and the control group. In the non-paretic limb, we observed deficits in PROM (*p* = 0.018) and AROM (*p* = 0.048), a lower average (*p* < 0.001) and maximum speed (*p* < 0.001) in FROM, worse proprioception (JPS, *p* < 0.001), and a lower number of repetitions in the step test (*p* < 0.001) compared to the control group. We also observed a decrease in the average isometric strength of the extensor (*p* < 0.001) and flexor (*p* = 0.040) muscles of the non-paretic knee joint compared to the CG. The balance assessment on a balance platform showed worse postural control in people after stroke in all tested conditions (eyes open and closed on a firm and foam surface; *p* < 0.001). (4) Conclusions: The non-paretic lower limb in stroke patients is characterized by limited ROM at the knee joint, reduced movement speed, decreased proprioception, weakness of the knee flexors and extensors, and resulting impaired balance. The deficits identified require improvement and should be considered when planning rehabilitation.

## 1. Introduction

A stroke is associated with a set of symptoms, including paresis and the functional limitation of the side of the body opposite to the lesion (paretic side, affected side) [1]. However, impairment on the ipsilateral body side also occurs, but the level of such deficits on the less-affected side (non-paretic, non-affected) has not yet been sufficiently described in functional terms [2,3]. As indicated by Scano et al. [4], although ipsilesional deficits are less severe than contralesional ones, they may negatively impact the performance of daily activities in people after a stroke. Therefore, understanding the role of the damaged brain hemisphere on motor control seems important and may help when planning the functional recovery of patients.

As we know, the cerebral hemisphere controls voluntary movements of the opposite side of the body through most of its fibers; however, a few proportions of the fibers also have innervations for same-side movements. This is probably one of the reasons for the occurrence of deficits on the non-paretic side of people after a stroke [1,4,5,6,7]. The presence of a bilateral component in motor control, supported by observations that a functional activation might have on bilateral expression in cortical areas, may suggest that each hemisphere plays a role in controlling the movements of both sides of the body [8,9]. It has also been suggested that the interconnection between the two hemispheres via the corpus callosum provokes the contralateral hemisphere to be involved in modulating the activation of the ipsilateral hemisphere [4,10]. Bagnato et al. [8]. suggested that in patients with hemispheric stroke, abnormal motor performances in the ipsilateral limbs may be due to cortical reorganization in the unaffected hemisphere, but some peripheral mechanisms may also play a role. We found some reports that described deficits (especially motor deficits) in the non-paretic limbs, but these data more frequently concerned the non-paretic upper limb [2,4,11,12,13,14]. Scano et al. [4] observed that the ipsilesional upper limb of post-stroke patients showed motor deficits in kinematic, dynamic, motor control, and energy consumption parameters during multi-joint movements. In a systematic review, Kitsos et al. [12] described evidence of abnormal patterns of movement and strength in the ipsilesional upper limb, indicating that these deficits can be associated with reduced functional capacity after a stroke and may impact patient outcomes. Pandian et al. [2] showed that the ipsilesional side of post-stroke subjects had significant motor deficits in terms of coordination, gross and fine motor dexterity, and muscle strength of the upper and lower extremities. Jeon et al. [11] indicated that there is sufficient evidence that motor function is abnormal on the non-paretic side of individuals following a stroke, as evidenced by muscle weakness. Other authors [13] indicate that the differences in the performance of non-paretic legs in patients after stroke compared to those of controls are due to the compensatory mechanisms observed, for example, during walking. Although impairment on the non-paretic side may be mild and unnoticeable, the asymmetry resulting from such weakness may lead to gait and balance disturbances during functional activities [1].

Correct muscle strength, balance (static and dynamic), proprioception, and joint mobility are essential for safe and independent mobility, including independent changes in body position, walking, and stair climbing. All these aspects should be assessed in stroke patients to properly plan their rehabilitation program, which aims to restore the patient’s maximum independence in everyday life. In scientific research, clinical assessments, and rehabilitation, less importance is usually attached to assessing the deficits occurring in the non-paretic limb. Moreover, in most studies, the attention is focused on comparing the disturbed functionality of the paretic limb with the fully functional limbs of healthy people [15] or with the ipsilesional one, commonly called “non-paretic” [16,17], which is not entirely reliable in people after a stroke, knowing that there are some deficits in the non-paretic limb. It is worth mentioning here that in terms of building static and dynamic efficiency for a person’s gait, the lower limb opposite to the paretic one will act as a kind of “flywheel”, hence the need for a detailed description of its functionality in hemiplegics.

Also, the literature analysis indicates that assessing deficits in the non-paretic lower limb in the early period after a stroke is not often described. Therefore, our research aims to assess the range of motion, movement speed, joint position sense, muscle strength, and static and dynamic balance in the non-paretic lower limb and compare it to a control group and the paretic lower limb.

## 2. Materials and Methods

### 2.1. Participants

Twenty-nine individuals (age: 52.9, SD = 7.8 years, 12 females, 18 right hemiparetic, weight: 83.6, SD = 14.3 kg, height: 171.6 SD = 8.7 cm) after stroke and twenty-nine similarly aged control individuals (age: 50.9, SD = 7.4 years, 14 females, weight: 81.6 kg, SD = 15.7, height: 173.8 SD = 9.9 cm) were recruited for the observational study (Table 1) [18]. Participants after a stroke were recruited from the Neurological Rehabilitation Department of Wiktor Dega Orthopedic-Rehabilitation Clinical Hospital, Poznań University of Medical Sciences. Written informed consent was obtained from all study participants after an explanation of the aims and methodology of this study. The study was conducted according to the Declaration of Helsinki and with the approval of the Bioethics Committee of the Poznań University of Medical Sciences (reference number 822/21).

The inclusion criteria of the study group were as follows: first stroke (confirmed by computer tomography (CT)), age ≤ 65 years old, time from stroke < 6 months, ability to stand independently for at least 5 min without an assistive device, ability to walk 5 m independently without any orthotic device or the help of another person, muscle strength on the Manual Muscle Test (MMT) of ≥4 in the paretic limb, spastic tension of the paretic lower limb according to Ashworth ≤1+, a Barthel Index score ≥ 85, and the ability to communicate and understand the tasks required in the study.

The exclusion criteria were the following: lack of active movement in the knee joint, neglect syndrome, sensorimotor aphasia, disorders in the field of vision, cognitive disorders that would make it impossible to understand commands, a lack of informed consent to participate in the study, other neurological diseases (such as MS, Parkinson’s disease, or neuropathies), fractures in the lower limbs which could affect the structure and function of the knee joint, and previous operations on the lower limbs.

The control group consisted of healthy volunteers with no prior history of trauma or neurological disease affecting the structure and function of the lower limb.

The sample calculation was carried out using PQStat (v1.8.6.122, Poznań, Poland). Data such as average values for the isometric strength of the extensor obtained for the first ten subjects were used to determine the required sample size. The alpha level was set at 0.05, and the power of the test (1-beta) was set at 0.8. The minimum required sample size was 22 subjects. Finally, we recruited 29 subjects for each group.

### 2.2. Testing Procedures

We assessed both lower limbs in the stroke group, presenting results for the non-paretic and paretic lower limbs. In the control group, we evaluated the left and right lower limbs. To objectify the assessment and exclude the potential influence of the dominant side on the results of the control group, we calculated the average score of the control group’s left and right lower limbs. This is presented in the Section 3 as the “control” limb and compared to the outcomes of the non-paretic and paretic sides of the stroke group.

#### 2.2.1. Assessment Using Wireless Motion Sensors

**Figure 1:**
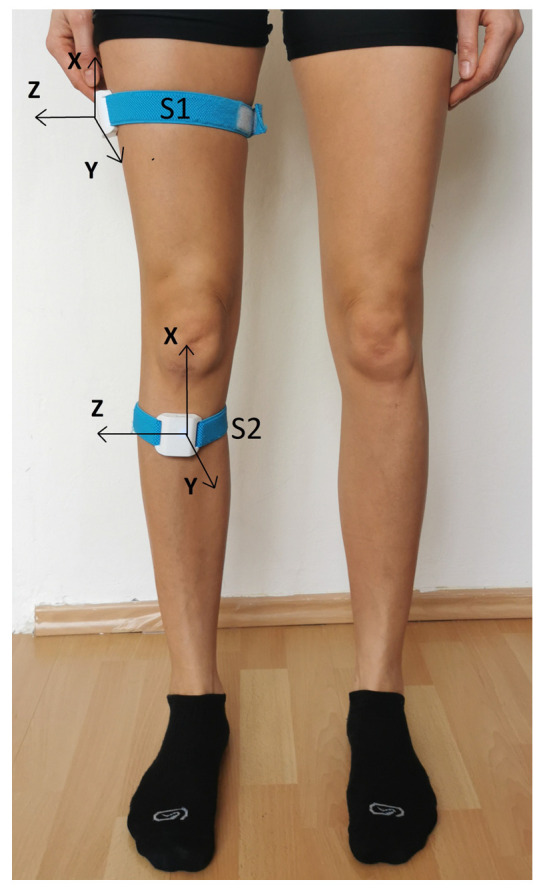
Placement of wireless sensors on the lower limb [19].

We used wireless motion sensors (Orthyo^®^ system, Aisens sp. z o. o., Poznań, Poland) connected to a mobile application installed on smartphones equipped with the Android operating system to assess the passive range of motion (PROM), active range of motion at any speed (AROM) and maximum speed (FROM) and proprioception (joint position sense, JPS). Before the study, each participant was registered in the Orthyo online panel. Then, two sensors were attached to each participant’s lower limbs using elastic Velcro straps. The first sensor (S1) was attached to the lateral surface of the thigh, 15 cm distal to the greater trochanter, and the second sensor (S2) was attached to the anterior surface of the tibia, 5 cm distal to the tibial tuberosity (Figure 1). Four sensors (2 per limb) and two smartphones were used. During the tests, the subject lay on their stomach with their lower limbs extended, their head in a neutral position, and their feet off the couch.

The Orthyo^®^ System uses three basic types of sensory data: velocity, acceleration, and magnetic field. The sensor collects raw sensory data, which are filtered, calibrated, and calculated in the estimation process by the sensor’s microchip. As a result, the sensor generates orientation and relative position. The location of the sensors was determined in a reference frame, the axes of which were positioned according to the East North Up (ENU) principle, where X points eastwards, Y points northwards, and Z points upwards. The estimation and calibration were performed based on estimators such as the Kalman filter, complementary filters, and supporting artificial intelligence algorithms. After the initial analysis, all calculated data were sent to the Orthyo application via low-energy Bluetooth, initiating the second data processing stage. At this stage, all the interdependencies between the sensors were calculated, yielding parameters that represented the movement of a knee joint (e.g., linear velocity, acceleration, and movement in space) [20]. The kinematic metrics were derived based on the difference between the orientations of the two IMUs (inertial measurement units). This difference was calculated using quaternion mathematics, which was then converted into angular values to represent the range of motion. The sensors were declared to have measurement conformity.

Depending on the test, selected parameters were recorded from those given below:

- Range of motion (degrees; °);
- Average angular velocity in the knee joint during the diagnostic examination (AVG speed, °/s);
- Maximum angular velocity during the test (MAX speed, °/s).

The examiner started recording the results in the application by simultaneously giving the “start” command and stopped the recording after the subjects completed the examination. Results were recorded separately for each limb.

##### Passive Knee Range of Motion (PROM)

The examiner stabilized the subject’s pelvis with one hand and passively flexed the knee joint with the other hand (until resistance occurred or the patient reported pain). The sensors recorded the range of motion, i.e., the difference between the initial and maximum angle in the knee joint.

##### Active Knee Range of Motion (AROM)

The examiner stabilized the subject’s pelvis with one hand. The subject was asked to perform maximum knee flexion at any speed after the “start” command, initiating the recording. The sensors recorded the range of motion, i.e., the difference between the initial and maximum angle in the knee joint.

##### Fast Active Knee Range of Motion (FROM)

The examiner stabilized the subject’s pelvis with one hand. The subject was asked to flex and extend the knee joint as quickly as possible after the “go” command was given to start the recording. In this test, the range of motion and the average and maximum angular velocity were recorded.

##### Proprioception of the Knee Joint (Joint Position Sense, JPS)

This test assessed the participant’s ability to reproduce a given position without a visual modality. The examiner passively flexed the subject’s knee joint to the selected position, then held the position for 5 s, asking the subject to remember it (without looking), and then straightened the knee joint. The subject was asked to reproduce the previously indicated position and give the command “stop” or “ok”. The application measured the achieved angle and calculated the difference between this angle and the set angle, which we analyzed. The sense of joint position was assessed in ranges at 80°. Before the test, the subjects did not know the values of the given angles.

#### 2.2.2. Force Measurement

The isometric strength of the extensor and flexor muscles of the knee was assessed using the Leg Force Feedback device. The patient was stabilized on a chair with their arms across their chest and the hip with knee joints flexed to 90°. The center of the dynamometer’s axis of motion was located near the lateral line of the knee joint, and the lever arm was attached to the ankle area. Analog torque signals were displayed and recorded on the screen for analysis. They were visible to the patient and were fed back during the measurement [21].

The subject was acquainted with the test methodology before starting the measurement. After familiarizing subjects with the equipment and procedure, they were asked to generate the maximum force in the knee joint and hold it for 10 s after the “start” command. The extensors (quadriceps muscle) and the knee flexors (the hamstring muscle groups) were assessed. The strength of both limbs was assessed independently.

The results were presented as follows:

- Maximal voluntary isometric contraction values—MVIC [Nm];
- Average values—AVG [Nm];
- Max moment—MM [Nm/s].

#### 2.2.3. Balance Assessment

##### The Step Test (ST)

In this test, the subject placed their foot on a 7.5 cm high step as quickly as possible within 15 s. The number of repetitions was measured. The measurement was performed twice for each limb. The average result from 2 measurements was analyzed [22].

##### Static Balance Assessment

Postural balance tests were performed using the HUMAC Balance System (CSMi). Before the test, the examiner input the patient’s data and information, including age and height, into the software. With the height and age of the individual entered, the device could adequately measure the displacements of the COG (center of gravity), which is located at approximately 55% of human height [23]. Also, the foot position on the platform was recorded (the middle of the heel lined up with the 7s on the horizontal axis, with medial malleolus at level C) because the HUMAC uses it to compute the test results.

**Figure 2:**
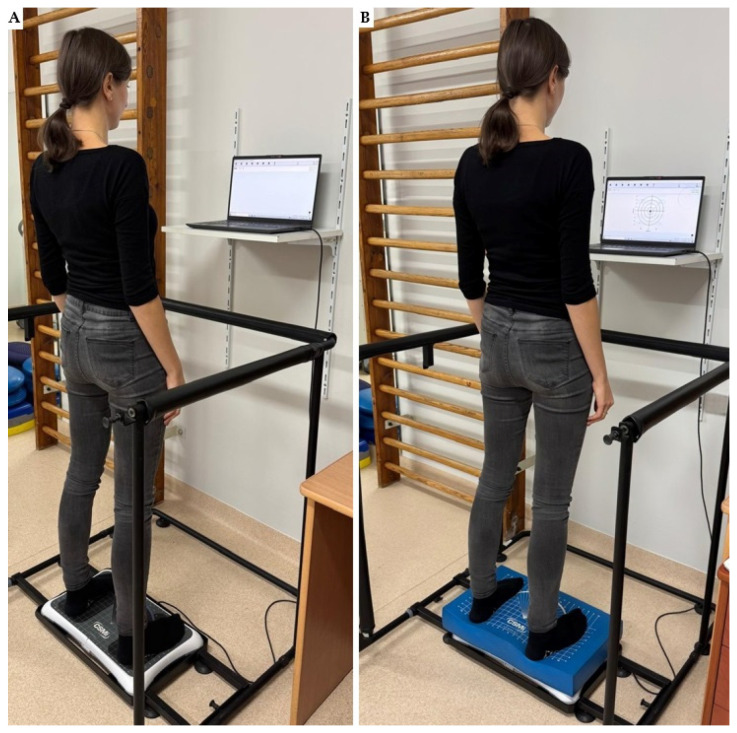
Participant performing the mCTSIB test: (**A**) standing on a firm surface; (**B**) standing on a foam surface.

During the balance tests, the participant stood barefoot on the HUMAC board with their feet parallel to each other, arms next to their sides, and eyes looking straight ahead (Figure 2). The subjects were asked to maintain a motionless upright position. The balance evaluation was performed using the modified Clinical Test of Sensory Integration and Balance (m-CTSIB). This modified version of the original CTSIB eliminates the visual conflict domain [23].

It consists of four conditions [23,24] as follows:

- Standing on a firm surface with the eyes open (EO)—the “standard” test condition where all three sensory systems (i.e., proprioception, vision, and vestibular) are available to assist in maintaining balance.
- Standing on a firm surface with the eyes closed (EC)—eliminates the visual input to evaluate vestibular and somatosensory inputs.
- Standing on a foam surface with the eyes open (EOF)—the visual and vestibular systems are available, but the proprioceptive system is compromised when the subject stands on a foam surface.
- Standing on a foam surface with the eyes closed (ECF)—the visual and proprioceptive systems are compromised, which allows the singular vestibular inputs to be evaluated.

According to the software evaluation protocol, all test times and position requirements were identical for each subject. The duration of the tests was 30 s each.

The test allowed us to obtain parameters characterizing the excursion of the center of pressure (COP) as follows:

- The total path length (P; cm);
- The average velocity (AV, cm/s).

### 2.3. Statistical Analysis

Data were analyzed using Statistica (v13.3.721.1) and PQStat (v1.8.6.122, Poznań, Poland). Demographic data are presented as the means, standard deviations (SD), median, minimum (min), and maximum (max). The Shapiro–Wilk test was used to assess the normality of the distributions in the test score. Student’s *t*-test for independent variables or the non-parametric Mann–Whitney U-test was used to compare the differences between demographic data and balance measurements. Differences between the non-paretic, paretic, and control limbs were assessed using one-way ANOVA with Fisher’s test as a post hoc assessment. *p*-values less than 0.05 were considered statistically significant.

## 3. Results

### 3.1. Assessment Using Wireless Sensors

Passive Knee Range of Motion (PROM)

**Figure 3:**
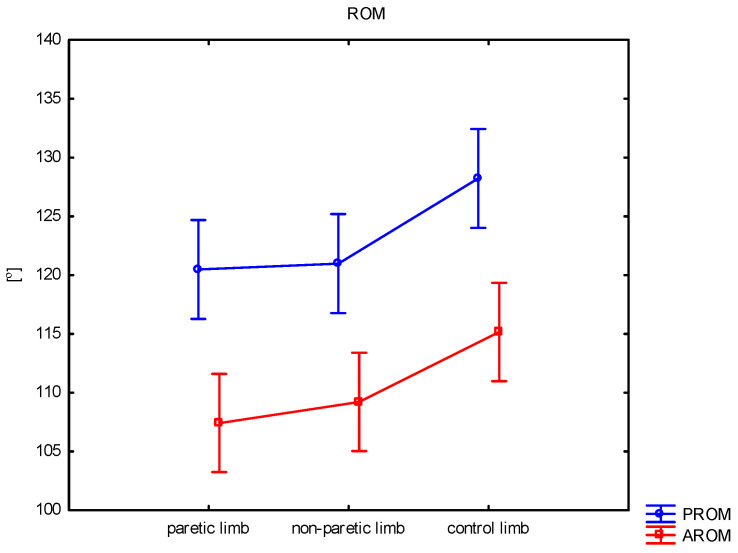
Comparison of passive (PROM) and active (AROM) range of motion (ROM) between paretic, non-paretic, and control limbs.

Table 2 and Figure 3 show the results of the passive range of motion assessments. There was a statistically significant difference between the results of the paretic and non-paretic limbs and the results of the control group. The average ROM for the non-paretic limb was 121.0° and was, on average, 7.2° lower than the results of the control group, while the average ROM for the paretic limb was 120.5° and was, on average, 7.7° lower than the results of the control limb (*p* = 0.011).

Active Knee Range of Motion (AROM)

The analysis of the results of active knee flexion indicates a statistically significant difference between the results of the non-paretic and paretic limbs and the results of the control group (Table 2 and Figure 3). The average ROM for the paretic limb was 107.4°. The results were, on average, 7.8° lower than the results of the control limb (*p* = 0.011), while the average ROM for the non-paretic limb was 109.2° and was, on average, 6.0° lower than the results of the control group (*p* = 0.048).

Fast Active Knee Range of Motion (FROM)

**Figure 4:**
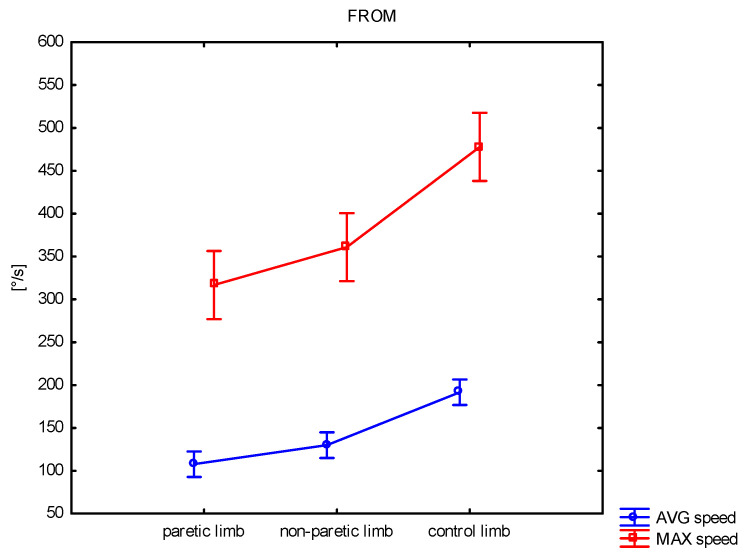
Comparison of average (AVG) and maximal angular speed between paretic, non-paretic, and control limbs.

The results obtained during the fast active knee flexion movement show significant differences between the groups regarding their average and maximum speed (Table 2, Figure 4). Average speed differed between the non-paretic and paretic limbs (*p* = 0.039) and between the non-paretic and paretic limbs and the control limb (*p* < 0.001; *p* < 0.001). The maximum speed differed between the non-paretic and paretic limbs and the control group, but at the same time, no difference was observed between the non-paretic and paretic limbs.

Proprioception of the Knee Joint (joint position sense, JPS)

**Figure 5:**
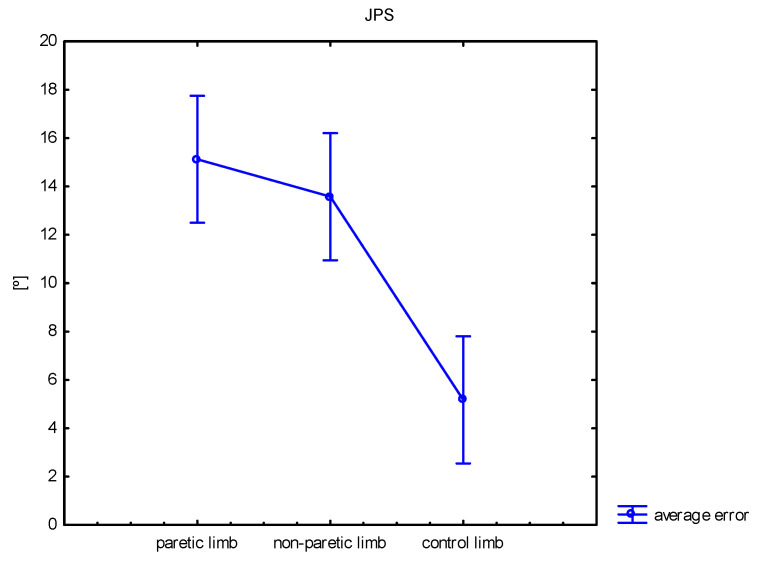
Comparison of the average error observed during the joint position sense measurement (JPS) between paretic, non-paretic, and control limbs.

The results obtained in the JPS test show significant differences between the groups (*p* < 0.001). The post hoc analysis indicated a significant difference between the non-paretic and control limbs (*p* < 0.001) and the paretic and control limbs (*p* < 0.001). The average error for the non-paretic limb was 13.6 ± 7.4; for the paretic limb, the average error was 15.1 ± 9.5; and for the control group, the average error was 5.2 ± 2.6. See Figure 5.

### 3.2. Force Measurement

#### 3.2.1. Extensors

Table 3 shows the results of measuring the isometric strength of the knee extensors obtained by stroke patients and the control group. Maximum (*p* < 0.001) and average voluntary contraction values (*p* < 0.001) and maximal moment results (*p* < 0.001) differed significantly between groups. The maximum strength was 7.8 Nm for the paretic limb, 9.8 Nm for the non-paretic limb, and 12.7 Nm for the control limb. Interestingly, the results of the non-paretic limb were significantly worse than those obtained from the control group.

#### 3.2.2. Flexors

**Figure 6:**
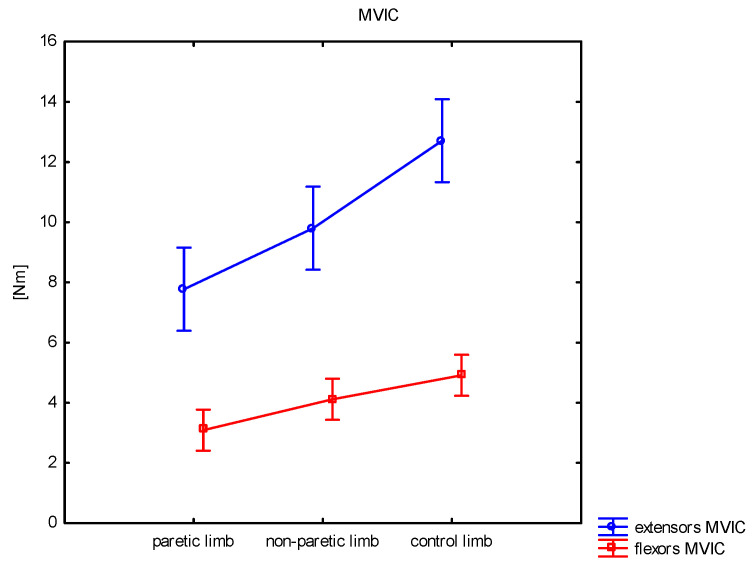
Comparison of maximal voluntary isometric contraction (MVIC) results for extensors and flexors between paretic, non-paretic, and control limbs.

Table 4 presents the measurements of the isometric strength of knee flexors obtained from patients after stroke and in the control group. Maximum and average voluntary contraction values and maximum moment scores differed significantly between the groups. In all parameters, significantly worse results were observed for the paretic limb than for the non-paretic and control limbs. Only AVG differed significantly between the non-paretic and control limbs. See Figure 6.

### 3.3. Balance Assessment

#### 3.3.1. The Step Test (ST)

Table 5 shows the results obtained in the step test from stroke patients and the control group. The mean score for the non-paretic limb was 12.0 ± 2.8, and the mean score for the paretic limb was 11.4 ± 3.0. Both results differed significantly from the control group’s outcome, which was 19.8 ± 2.7.

#### 3.3.2. Static Balance Assessment

We observed significantly worse results in people after stroke than in the control group, regardless of whether the examination was performed with the eyes open or closed and on a firm or foam surface. In all tests, stroke survivors had longer total distances and higher average speeds. Detailed data are presented in Table 6.

## 4. Discussion

After a stroke, one of the main goals of rehabilitation is to regain the ability to walk independently. However, climbing stairs is also essential to everyday mobility and significantly correlates with free physical activity in stroke survivors (stairs require additional strength, coordination, and balance concerning walking on the ground) [25]. Therefore, correct ranges of motion in both lower limb joints, muscle strength, coordination, and balance are needed to perform the abovementioned basic daily activities independently and safely. Many patients experience deficits in the above functionalities after a stroke, and data from the literature indicate that such deficits occur not only in the paretic limb, which is usually thoroughly assessed [2,3]. Therefore, there is a need to carefully investigate deficits occurring in the non-paretic limb, as this may also impact the daily mobility of stroke patients.

The lower limb joints’ range of motion (ROM) may be limited in patients after a stroke [26]. In our study, we focused on assessing the range of motion of the knee joints. We noticed deficits in the passive and active range of flexion motion in the knee joints in both limbs in stroke survivors compared to the control group. Many studies conducted among healthy volunteers have shown that, compared to walking on a level surface, a greater range of knee flexion angles is required when climbing stairs [27,28,29]. Therefore, regaining a normal range of motion for stroke survivors is essential for independent, safe, and proper locomotion at home and in the community. Furthermore, limited ROM in the hip, knee, and ankle joints not only makes walking or climbing stairs difficult, but patients may also have difficulty with activities such as dressing, using the toilet, bathing, picking up objects, squatting, tying shoelaces, and clipping nails [30]. Studies measuring joint angles while walking, climbing stairs, and standing up in healthy individuals have shown that hip flexion of 80°, knee flexion of up to 110°, and ankle dorsiflexion of 26° are required to perform these activities. However, the results of Hyodo et al. [30] indicate that other functional activities require more extreme joint angles than walking, climbing stairs, or standing up. Their study showed that the mean maximum knee flexion angle in ADL activities exceeded 110°. The average maximum knee flexion exceeded 140° during trunk rotation while crouching and exiting the bath with the dominant foot. Therefore, these movements require more knee flexion than is needed for walking, climbing the stairs, or standing up. It is worth asking the following question: which limb of a stroke patient is dominant? If the non-paretic lower limb is a dominant limb, insufficient range of motion in this limb may affect the ability to perform the indicated activities. If the dominant limb is paralyzed, the prognosis for regaining independence in walking may be significantly worse. Rowe et al. [28] suggested that 110° of flexion would seem a suitable goal for the rehabilitation of motion in the knee because gait and slopes require less than 90° of knee flexion, stairs, and chairs require 90–120° of flexion, and a bath requires approximately 135° of flexion. To summarize, limiting the range of motion in the lower limb joints may affect the performance of everyday activities. In our study, we observed deficits in the range of movement in both knee joints in stroke patients compared to the control group; therefore, we suggest that the assessment of the range of motion and exercises to improve this should concern not only the paretic lower limb but also the non-paretic lower limb, which is often ignored during rehabilitation.

Movement speed in stroke survivors is usually measured during gait tests and is expressed as walking speed (usually without distinguishing between the speed of movement of each limb) or during reaching movements [31,32]. Data from the literature indicate a significant deterioration in walking speed in people after a stroke compared to healthy controls. In our study, the methodology for assessing the speed of lower limb movement was different and allowed for an independent assessment of the velocity of motion of the non-paretic and paretic limbs. In the prone position, such as during ROM testing, we measured the average and maximum velocity during the knee flexion and extension movement (FROM). Interestingly, although the average speed of movement in the non-paretic limb was significantly higher than in the paretic limb, it was still significantly lower than in the control group. However, maximum speed did not differ in the paretic and non-paretic limbs, but both results were significantly worse than those in the control group. Both results again indicate the presence of deficits in the non-paretic limb and the need for a thorough assessment of this limb when planning rehabilitation. In hemiparetic patients, the control of the velocity of a single joint of the paretic limb may be complicated by spasticity-related inappropriate muscle activity. However, it is worth noting that many everyday activities, such as driving a car, stabilizing oneself while riding a bus, and taking a step after tripping to avoid falling, require quick movements of both lower limbs. As Hammerbeck et al. [33] indicated, the current clinical guidelines do not emphasize the need to train patients at various movement speeds, and there are limited studies investigating how movement speed during training affects learning after stroke. On the other hand, Liang et al. [34] indicated that for successful social locomotion, the ability to modulate (increase and decrease) walking speed is more important than continuous walking at a constant speed. Therefore, we believe that assessing the speed of movement and the ability to perform movements at any and maximum speeds could help determine rehabilitation goals and should also be applied to the non-paretic side of the body.

The sensory-motor dysfunction in stroke patients includes muscle weakness, abnormal muscle tone, and proprioception deficits. Proprioception plays an important role in maintaining dynamic joint stability, inducing normal movements, and protecting joints from external damage. Independent, safe walking requires adequate “proprioceptive efficiency” in both lower limbs. As Yang et al. [35] suggested, if a stroke degrades proprioception, it could also undermine muscle strength, normal muscle tone, posture control, protective reflex ability, and joint motor ability. Safe walking and stair climbing are associated with good proprioception. In studies assessing the range of motion needed to walk, climb, and descend stairs, the results often vary depending, for example, on the walking phase and height of the steps (on average, from 60 to 120°) [28,29,30]. Therefore, we decided to choose the 80° knee flexion range to assess proprioception (joint position sense) deficits in stroke survivors. Our observations show that proprioception deficits affect not only the paretic side but also the non-paretic limb. Stroke patients achieved a greater error during the tests performed in the lower non-paretic limb than the control group. The error for the paretic limb was also higher than the error of the control group. In the study by Hwang et al. [36], a significant proprioception deficit was observed not only in the paretic knees of both right and left hemiplegia patients but also in the non-paretic knee of right hemiplegia patients. However, these researchers evaluated the passive angle reproduction of 30° and 60° knee flexions. Our previous work discussed this topic in more detail [19].

Adequate muscle strength in both lower limbs is essential for independent walking and is associated with the ability to perform many activities of daily living (ADL) in stroke patients [37]. Wang et al. [38] indicated that knee extensor strength of the non-paretic leg is the most important determinant of exercise capacity in community-dwelling stroke survivors. Therefore, loss in muscle strength is an important factor affecting recovery after a stroke and is one of the barriers to reaching full independence [39,40]. According to reports [21], the mechanisms of reduced muscle strength can be classified as primary (resulting from muscle dysfunction) or secondary, resulting from a number of factors, including spasticity and disuse. Our study assessed the isometric strength of the knee flexor and extensor muscles in the subacute period after a stroke, showing that deficits in muscle strength also occur in the non-paretic lower limb. The maximal and average voluntary knee extensor contractions performed for the non-paretic limb were higher than in the paretic limb but smaller than in the control group. In the flexor examination, the non-paretic limb also had significantly lower AVG than the control group, while MVIC did not differ significantly. A limitation of our study is that we only assessed the strength of the knee flexors and extensors. Other authors who assessed other muscle groups in stroke patients also indicated decreased muscle strength on the non-paretic side of the body compared to healthy individuals [2,40,41,42]. Pandian and Arya [2] showed that all the muscle groups, including knee flexors and extensors of the less-affected lower extremity, were weaker than the controls. The researchers used Manual Muscle Testing (MMT) to assess participants. Dorsch et al. [42] measured the maximum isometric strength of 12 muscle groups using hand-held dynamometry. They concluded that the stroke participants’ intact lower limbs (non-paretic) were significantly weaker than the control participants for all muscle groups (for the extensors and flexors of the knee joint as well) except for the ankle invertors. Dengiz et al. [40] showed that the muscle strength of stroke individuals’ unaffected side, including total muscle strength in the lower and upper extremities, grip strength, and trunk extensor muscle strength, decreased dramatically compared to the healthy group. Our research, alongside that of others, indicates the need to use exercises to strengthen the paretic and non-paretic limb muscles, which should affect functional recovery after stroke.

It is reported that about 83% of stroke survivors may suffer from balance impairment [43], which directly affects autonomy, a lower level of activity, participation, and quality of life [44]. Many tests that assess balance and fall risk do not independently assess the paretic and non-paretic limbs. Such tests include commonly used methods such as the Tinetti scale, the Berg balance scale, the “get up and go” test, the functional reach test, the dynamic gait index, the four-square step test, as well as many tests performed on balance platforms. An interesting test that allows for the independent assessment of the paretic and non-paretic limbs in the context of evaluating dynamic balance is the step test. Our study assessed balance using a balance platform (static assessment) and a step test (dynamic assessment). On the balance platform, we observed significantly worse results in people after a stroke in than the control group in all tested conditions (eyes open and closed on a firm and foam surface). In the EO test, all three sensory systems (i.e., proprioception, vision, and vestibular system) were available for maintaining balance. In the second condition, the eyes were closed, causing the temporary elimination of visual feedback, thus increasing the dependence on the proprioceptive and vestibular systems. Since proprioception is used more for maintaining balance than the vestibular system, it can be assumed that this condition largely measures the contribution of proprioception to balance. In the third condition, when the visual and vestibular systems were available, and the proprioceptive system was compromised by standing on foam, the difficulties in maintaining a stable posture related to the visual system, given its preference over vestibular feedback for balance control. In the fourth condition, the eyes were closed, and the person stood on the foam. Therefore, it is assumed that the vestibular system plays the leading role in maintaining balance as the primary source of sensory input [23,24]. In all tests, participants after a stroke had a longer total path length and a higher average velocity, which indicates poorer balance control compared to healthy controls. The results indicating balance deterioration in people after a stroke are consistent with the observations of other authors. Awosika et al. [45] found significantly greater postural instability across all conditions in chronic stroke survivors compared to normative data. In addition, they found an increased reliance on visual and somatosensory systems in chronic stroke survivors in conditions two and three of the mCTSIB and higher sway velocity index values in condition four when both were absent or perturbed [45]. Peurala et al. [46] measured static balance while standing on a force plate, concluding that patients with right or left hemiparesis sway more than healthy subjects. Wang et al. [47] also observed that stroke patients exhibited decreased postural stability during quiet stances, especially under non-vision conditions. The second test that we used to assess balance was the step test, originally developed as a dynamic standing balance test after a stroke. As Mercer et al. [48] indicate, during the placement of individual steps with the paretic foot, the paretic lower limb must move quickly in flexion and reverse the direction of movement. When taking steps with the non-paretic foot, the paretic lower limb must have a stable extension and support the entire body weight. However, as we also noticed in the opposite situation, the non-paretic limb must be stable when the patient performs a step with the paretic limb. In our study, the stroke group performed an average of 11.4 ± 3.0 repetitions with the paretic limb, 12.0 ± 2.8 with the non-paretic limb, and the healthy subject performed 19.8 ± 2.7 repetitions. Hong et al. [49] observed that the mean ST scores were 8.1 ± 4.1 and 11 ± 4.2, respectively, with the paretic and non-paretic limbs, but the participants in this study were recruited on average 5.9 years post-stroke and were all community-dwelling and at least modestly ambulant. The average result for the right limb in the control group was 18.7 ± 4.0, and for the left limb was 18.6 ± 4.0. In the study by Thilarajah et al. [17], the median scores for paretic and non-paretic ST were eight and nine repetitions, respectively, but the mean age of the subjects was higher, and the time since stroke was shorter. These researchers observed that the non-paretic ST was better associated with the balance variables compared with the paretic ST and concluded that movement-induced perturbations may be greater when patients balance on their paretic limb (during the non-paretic ST), which, in turn, may require greater balance to maintain equilibrium. They indicated that individuals might also favor their non-paretic leg when standing so that their static standing position is affected when their non-paretic leg is compromised. They also indicated that both STs were associated with physical function, gait speed, TUG scores, and future falls. However, the researchers did not compare the results to those of a control group.

### Limitations

In this study, we propose a simple set of tests showing the differences between non-paretic and paretic limbs and the results of a control group. We are aware that in everyday physiotherapy practice, not everyone can routinely assess stroke patients using wireless motion sensors, a balance platform, or measure isometric strength with the necessary equipment. However, the parameters we examined can mostly be assessed using traditional methods, such as a goniometer, MMT test, or functional tests.

## 5. Conclusions

Our research indicates that examined functionalities of “non-paretic” lower limbs, such as knee range of motion, movement speed in the knee joint, knee joint position sense, and knee extensors and flexors muscle strength, differ significantly from healthy limbs. Determining the precise cause of these differences examined is still difficult. Deficits in paretic limbs may contribute to the occurrence of limitations in the functioning of the non-paretic limb, as we indicated by the step test results and static balance test on the balance platform. A lack of stability, appropriate range of motion, or strength of the paretic limb may also cause compensatory patterns to appear in the non-paretic limb during everyday activities such as walking or climbing stairs.

Our results indicate the critical role of rehabilitation, including improving the knee range of motion, muscle strength of the knee extensors and flexors, speed, and coordination not only in the limbs directly affected by the stroke but also in the non-paretic limbs (non-affected, non-involved, and ipsilateral). Additionally, during the assessment of stroke patients, it is worth remembering that non-paretic limbs should not be used as a reference point for the “normal” range of motion, proprioception, speed of movement, or strength of the paretic side.

## Figures and Tables

**Table 1:** Demographic data of the study group and the control group [19].

Variable		Stroke Group	Control Group	*p*
age [year]	mean ± SD median min–max	52.9 ± 7.8 53.0 39.0–64.0	50.9 ± 7.4 51.0 37.0–65.0	0.315
body mass [kg]	mean ± SD median min–max	83.6 ± 14.3 81.0 58.0–112.0	81.6 ± 15.7 82.0 60.0–120.0	0.608
height [kg]	mean ± SD median min–max	171.6 ± 8.7 173.0 152.0–186.0	173.8 ± 9.9 175.0 159.0–195.0	0.386
BMI	mean ± SD median min–max	28.5 ± 5.5 28.1 19.4–43.8	27.0 ± 4.4 26.5 19.2–36.0	0.236

*t*-Student’s test.

**Table 2:** The results obtained using wireless motion sensors (PROM, AROM, FROM, and JPS tests).

Variable		Paretic	Non-Paretic	Control	*p* ^1^	P vs. N	P vs. C	N vs. C
PROM (°)	mean ± SD median min–max	120.5 ± 11.5 119.5 96.0–146.2	121.0 ± 13.0 121.3 95.7–147.4	128.2 ± 9.4 129.2 112.4–143.9	0.018	0.866	0.011	0.018
AROM (°)	mean ± SD median min–max	107.4 ± 13.3 109.0 83.5–138.9	109.2 ± 12.4 109.4 86.7–133.5	115.2 ± 7.2 117.5 103.2–126.4	0.028	0.548	0.011	0.048
FROM (°)	mean ± SD median min–max	107.8 ± 13.1 110.5 83.2–130.8	110.2 ± 12.3 110.1 79.2–132.9	111.5 ± 8.4 112.2 95.5–128.3	0.456	-	-	-
FROM AVG speed	mean ± SD median min–max	107.6 ± 40.8 103.4 15.5–213.1	129.8 ± 37.0 117.2 62.5–214.8	191.7 ± 43.1 178.3 133.0–282.1	<0.001	0.039	<0.001	<0.001
FROM MAX speed	mean ± SD median min–max	316.7 ± 123.2 293.4 49.9–540.1	360.9 ± 104.1 346.3 109.0–588.0	477.9 ± 93.8 482.6 318.1–701.0	<0.001	0.122	<0.001	<0.001
JPS 80°	mean ± SD median min–max	15.1 ± 9.5 13.5 2.2–42.6	13.6 ± 7.4 12.3 0.0–32.0	5.2 ± 2.6 5.2 1.1–9.8	<0.001	0.411	<0.001	<0.001

^1^ ANOVA; P—paretic limb; N—non-paretic limb; C—control group.

**Table 3:** Results of measuring the isometric strength of the extensor knee muscles.

Variable		Paretic	Non-Paretic	Control	*p* ^1^	P vs. N	P vs. C	N vs. C
MVIC [Nm]	mean ± SD median min–max	7.8 ± 3.9 6.6 0.4–15.2	9.8 ± 3.1 9.5 5.3–17.6	12.7 ± 4.1 12.1 6.0–24.7	<0.001	0.042	<0.001	0.004
AVG [Nm]	mean ± SD median min–max	6.3 ± 3.2 5.3 0.4–12.2	8.0 ± 2.6 8.2 3.4–12.7	11.1 ± 3.6 10.2 5.4–20.7	<0.001	0.042	<0.001	<0.001
MM [Nm/s]	mean ± SD median min–max	65.9 ± 33.8 56.2 3.6–127.9	84.1 ± 26.8 86.0 36.0–133.6	116.8 ± 37.9 107.3 56.6–218.2	<0.001	0.039	<0.001	<0.001

^1^ ANOVA; P—paretic limb; N—non-paretic limb; C—control group.

**Table 4:** Results of measuring the isometric strength of the knee flexors muscles.

Variable		Paretic	Non-Paretic	Control	*p* ^1^	P vs. N	P vs. C	N vs. C
MVIC [Nm]	mean ± SD median min–max	3.1 ± 2.0 3.0 0.0–7.2	4.1 ± 1.6 3.9 1.7–7.5	4.9 ± 1.9 4.4 2.0–9.7	0.001	0.037	<0.001	0.104
AVG [Nm]	mean ± SD median min–max	2.4 ± 1.7 2.1 0.0–5.9	3.3 ± 1.3 3.1 1.5–5.9	4.2 ± 1.6 4.0 1.7–7.9	<0.001	0.029	<0.001	0.040
MM [Nm/s]	mean ± SD median min–max	25.5 ± 17.5 21.1 0.0–62.2	35.1 ± 14.2 32.5 15.4–62.2	43.4 ± 17.5 39.8 18.0–83.5	<0.001	0.030	<0.001	0.060

^1^ ANOVA; P—paretic limb; N—non-paretic limb; C—control group.

**Table 5:** The comparison of results obtained in the step test between groups.

Variable		Paretic	Non-Paretic	Control	*p* ^1^	P vs. N	P vs. C	N vs. C
Step test	mean ± SD median min–max	11.4 ± 3.0 11.3 5.0–16.8	12.0 ± 2.8 12.3 5.8–16.3	19.8 ± 2.7 20.3 14.5–24.5	<0.001	0.434	<0.001	<0.001

^1^ ANOVA; P—paretic limb; N—non-paretic limb; C—control group.

**Table 6:** Comparison of the results obtained in the static balance assessment using the m-CTSIB test (EO—eyes open on a firm surface; EC—eyes closed on a firm surface; EOF—eyes open on a foam surface; ECF—eyes closed on a foam surface).

Variable			Study Group	Control Group	*p* ^1^
EO	(cm/s)	mean ± SD median min–max	29.7 ± 18.9 26.0 13.2–111.9	16.6 ± 6.5 14.3 8.8–37.8	<0.001
	(cm)	mean ± SD median min–max	1.0 ± 0.6 0.9 0.4–3.7	0.5 ± 0.2 0.5 0.3–1.3	<0.001
EC	(cm/s)	mean ± SD median min–max	54.3 ± 37.4 42.4 16.0–206.2	29.1 ± 17.3 24.2 13.0–101.0	<0.001
	(cm)	mean ± SD median min–max	1.8 ± 1.2 1.4 0.5–6.9	1.3 ± 1.7 0.8 0.4–9.8	<0.001
EOF	(cm/s)	mean ± SD median min–max	68.6 ± 41.5 60.0 31.0–188.2	38.2 ± 13.4 34.7 19.1–75.0	<0.001
	(cm)	mean ± SD median min–max	2.3 ± 1.4 2.0 1.0–6.3	1.3 ± 0.4 1.2 0.6–2.5	<0.001
ECF	(cm/s)	mean ± SD median min–max	187.4 ± 121.0 139.0 84.0–662.1	106.9 ± 40.2 105.7 55.6–209.2	<0.001
	(cm)	mean ± SD median min–max	6.2 ± 4.0 4.7 2.8–22.1	3.6 ± 1.4 3.5 1.9–7.0	<0.001

^1^ Mann–Whitney test.

## Data Availability

The data analyzed during the current study are available from the corresponding author upon reasonable request.
